# Extortion experiences of recent adult immigrants from Latin America: self-reported prevalence, associated costs, and current mental health

**DOI:** 10.1186/s40621-024-00524-2

**Published:** 2024-09-05

**Authors:** Laura Vargas, C. Neill Epperson, Therese S. Richmond, Shadi Sharif, Lily Berkowitz, Zachary Giano, Stephen Hargarten, Mark Ungar, Eugenio Weigend-Vargas, Joseph Sakai

**Affiliations:** 1 University of Colorado Anschutz Medical Campus, Aurora, USA; 2 University of Pennsylvania School of Nursing, Philadelphia, USA; 3 Medical College of Wisconsin, Milwaukee, USA; 4 City University of New York, New York, USA; 5 University of Michigan-Ann Arbor, Ann Arbor, USA; 6 Department of Psychiatry, University of Colorado SOM, Aurora, CO USA

**Keywords:** Extortion, Violence, Traumatic exposure, Latinx immigrants, Mental health, PTSD

## Abstract

Violence across Latin America is an increasingly important factor influencing migration to the US. A particular form of violence that is experienced by many Latinx migrants is extortion. This research analyzes the extortion experiences of Latinx immigrant adults arriving at the US southern border and the impact these experiences have on mental health. We find that on average, participants paid $804 in extortion during their migration. The most common perpetrators of extortion in our study were police followed by immigration officials throughout Latin America. Pregnant participants were less likely to experience extortion and adults traveling with children were more likely to be extorted. Participants who were extorted for money reported significantly greater severity of post-traumatic stress disorder (PTSD) symptoms compared to those who were not extorted. This research is the first of its kind to analyze extortion experiences among Latinx immigrants to the US, quantifying the prevalence, amounts paid, countries where extortion occurs, and perpetrators of extortion. In addition, extortion experiences are associated with negative effects on the mental health of newly arrived Latinx immigrants to the US. Based upon these findings, we recommend that extortion should be considered a significant stressor in the migrant experience, particularly for those adults traveling with children.

## Introduction

Migration from Latin America to the United States (US) has been on the rise in recent decades due to regional violence, political and economic instability, as well as various changes in US immigration policies (e.g. policies allowing certain migrants to reunify with family members in the US). As a result, the US Latinx population has increased nearly nine-fold since the 1960s, with an expected total growth to 107 million by 2065 (Colby and Ortman 2014). Although previously Latinx migrants tended to be male and working age, increasingly families, women, and children are making the journey to the US border (UNHCR The UN Refugee Agency 2016). Considering the large-scale demographic changes of migration to the US, improved understanding of the current Latinx migrant population is a research priority.

Violence, particularly due to the growing activity of organized crime networks, is a significant contributing factor to migration (Guerrero-Gutiérrez 2011; Muggah and Aguirre 2018). One critical form of violence motivating migration from Latin American countries is extortion (Contreras 2014). For instance, Rios found every case of extortion is associated with county-level migration outflows in Mexico by 13.03 per 100,000 people (Contreras 2014). Additionally, because the migration journey to the US requires resources (e.g. savings to pay formal and informal migration means), Latinx migrants are especially vulnerable to extortion, theft, human trafficking, and other crimes en route (Vogt 2013). Work in other countries (e.g. Indonesia, Libya) suggests extortion occurs across a range of immigrant populations and situations (Phillips and Missbach 2017). In Africa, extortion is often committed by police, immigration officials, or other law enforcement personnel (Gaibazzi 2017). A few studies provide in-depth ethnological accounts of extortion, kidnapping and other violence experiences of Central American migrants in Mexico (Vogt 2013). These qualitative studies suggest that extortion is a common experience during migration and that migrants are targeted, by force, to pay any money they carry or ask family members to make extortion payments from abroad (Vogt 2013). What these studies lack is information on the prevalence of extortion experiences, amounts paid in extortion, types of extortion perpetrators and countries.

Experiences of extortion may have lasting impacts on an individual’s mental health. For example, O’Connor’s pilot study found extortion or robbery experiences within Mexico were associated with depression, anxiety, and Post-Traumatic Stress Disorder (PTSD) symptoms (O’Connor et al. 2014). To our knowledge, previous studies have not provided a clear picture of the prevalence of extortion as a traumatic experience among Latinx immigrants to the US. This study also builds on prior studies to examine extortion as a traumatic experience related to symtoms of depression, anxiety, and PTSD among a sample of newly arrived immigrants to the US from Latin American countries.

This analysis focuses on understanding the prevalence of extortion experiences. However, the question of what constitutes extortion among migrant samples varies across the literature. Prior research suggests that not all interactions between migrants and smugglers or “facilitators” may be exploitative (Sanchez 2017). We count exortion experiences in this study as the experiences in which participants told us they were asked/coerced to pay money through the (implicit or explicit) threat of use of force by potential perpetrators. Further we describe the types of extorters that participants encountered to understand how migrants may perceive these experiences as exploitative, or not.

Increased research on the prevalence and degree to which extortion occurs in Latinx migrant populations is needed. The aims of this initial study were to describe the (1) prevalence of extortion for money amongst individuals recently immigrating to the US from Latin America; (2) characterize the extortion experiences including countries where it occurs, perpetrators and amounts; (3) test for differences between those immigrants experiencing vs. those not experiencing extortion (demographics, approximate distance traveled, mode of transportation utilized, as well as current mental health, and self-rated general health and quality of life among Latinx immigrants in transit to the US).

## Methods

Research Design: This study is a cross-sectional study of migrants recruited at the southern border of the US.

Sample description: The parent study has been ongoing since June of 2022, but a set of extortion related questions were added to the battery on February 13th, 2023. Because US immigration policy changed on May 11th 2023 with the end of Title 42 (Long C. AP News 2023) these analyses use the subsample interviewed between February 13th through May 10th 2023.

Inclusion/exclusion criteria: Adults 18 years of age and older, migrating from a Latin American or Caribbean country and entering through the US southern border were recruited. All participants entered the US within the last 14 days of the time of study enrollment. We excluded potential participants who did not speak or understand Spanish or English.

Recruitment methods: We approached potential participants at a humanitarian respite center in southern Texas, explained the study protocol, and obtained verbal consent from eligible and interested participants. Structured survey interviews were conducted by a bi-lingual researcher (LXV) and surveys were stored in REDCap. We provided a $50 gift card to participants for reimbursement for their time.

Data Collection: Overall, the complete survey that includes the measures mentioned in the methods section and others that are not a part of this analysis takes about 45 min of participant’s time to complete. A prior publication by Vargas et. al. (2024) provides a detailed description of participant recruitment and data collection processes for this study (Vargas et al. 2024).

### Survey instruments

Extortion: Questions about extortion were adapted from the Harvard Trauma Questionnaire but conflated extortion and robbery (HTQ; Peruvian Version) (Suarez 2013; Berthold et al. 2019). On February 13th, 2023 we added an item to specifically assess, “Were you extorted for money?” (in these analyses, we focus on those extorted for money). Additional questions included, “What type of individual(s) extorted you? (choose all that apply)”; “How much money did you have to pay in extortion?”; “In which country did this occur? (choose all that apply)”. When asked about their extortion experiences, participants often described in detail the circumstances of these experiences. We provide an example of a common extortion experience in our discussion section. The current study also utilizes information on participant demographics and questions about their migration journey.

Mental Health: We used the Adverse Childhood Experiences (ACEs) to assess for number of childhood traumatic experiences in seven different categories of exposure (Felitti et al. 1998). We assessed current/recent mental health symptoms of depression, anxiety, and PTSD using PHQ-9 (Kroenke et al. 2001), GAD-7 (Spitzer et al. 2006), and PCL-5 (Blevins et al. 2015) instruments, which have been widely validated and used in Spanish speaking and Latinx populations (Cantonis and Osborn 2017; Caplan and Buyske 2015; Abaya et al. 2021; Ramos et al. 2017; Sahbaz 2024; Martinez et al. 2023). Finally, we asked participants a question about self-rated mental health, “How would you rate your mental health?,” and separate questions rating overall health and quality of life using a Likert type scale for each. (excellent, very good, good, fair, or poor).

Currency conversion: Extortion values provided in other currencies were converted to the US Dollar (USD) using the exchange rates of each currency on February 13th, 2023.

Analytic Strategy: Descriptive statistics were generated for all study variables for the sample, those who were extorted, and those not extorted. T-tests, chi-square, log likelihood ratio, and Mann Whitney tests were used to detect differences between those who were extorted and those not extorted (depending on the variable type and skew of distribution).

## Results

Our sample consists of 85 Latinx immigrant adults. We approached a total of 113 potential participants during recruitment, of which 85 agreed to participate in this study; an acceptance rate of 75.2%. Those who were not enrolled in the study referenced different reasons (i.e. they were too tired to participate, did not want to participate, or did not have enough time to participate).

Table 1 presents demographic characteristics of our sample, comparing differences between two groups: those who were extorted for money (n = 67; 78.8%) of which 65 reported an amount for an average amount paid in extortion of $804 and those who were not extorted (n = 18; 21.2%). Males and females were not significantly different in their extortion experiences. Although individuals who were extorted for money were slightly older than those who were not (32.4 vs 29.3 years), this difference was not significant (*p* = 0.13). Other demographic characteristics such as marital status, monthly income, years of education, and employment were not different between groups extorted and not extorted for money. However, individuals from a South American country were more likely to be extorted for money than individuals from Central America/Mexico/Caribbean. Women who were pregnant or men traveling with a pregnant spouse were significantly less likely to be extorted for money. However, adults traveling with children were more likely to experience extortion. (see Table 1).

**Table 1:** Demographics and migration characteristics

	Full Sample (n = 85)		Not extorted (n = 18)		Extorted (n = 67)		Sig.
	n/μ	%/SD	n/μ	%/SD	n/μ	%/SD	
Female	59	69.40	15	83.3	44	65.7	0.149^a^
You/your partner currently pregnant	12	14.1	7	38.9	5	7.5	**0.004**^a^
Employed before leaving home country	76	89.4	14	77.8	62	92.5	0.070^a^
From a South American country (Compared to all others)	53	62.4	5	27.8	48	71.6	** < 0.001**^a^
Marital status							0.430^b^
Divorced	3	3.5	1	5.6	2	3.0	
Married	11	12.9	4	22.2	7	10.4	
Separated	6	7.1	2	11.1	4	6.0	
Single	65	76.5	11	61.1	54	80.6	
No. mths employed in last year	8.2	4.3	7.2	5.1	8.5	4.0	0.250^c^
Age	31.7	7.5	29.3	8.4	32.4	7.2	0.130^c^
Years of schooling	10.5	3.6	9.1	3.7	10.9	3.5	0.065^c^
Monthly Income ($)	247.0	217.7	268.7	243.7	240.7	211.3	0.695^d^
Traveling with kids (yes)	61	71.8	8	44.4	53	79.1	**0.004**^a^
Forced displacement (yes)	33	38.8	5	27.8	28	41.8	0.279^a^
Airplane (yes)	15	17.6	5	27.8	10	14.9	0.204^a^

Bold values are significance by* p* < 0.05

a = Chi-Square, b = log likelihood ratio, c = t-test, d = Mann Whitney

Table 1 also compares migration journey characteristics and mental health of the extorted and not extorted groups. Immigrants from a South American country were extorted at a higher rate than individuals from all other nations. There were no differences in extortion by reason for migration or mode of transportation. Table 2 outlines specific demographics of the extorted group. Participants could indicate one or more countries where the extortion occurred, extortion occurred most frequently in Mexico (77.6%) and Guatemala (67.2%), followed by Colombia (22.4%), Nicaragua (20.9%), and Honduras (17.9%; see Table 2).

**Table 2:** Extortion characteristics

		Extorted (n = 67)	
		n/μ	%/SD
Avg extortion amount paid (USD)*		804.68	980.82
Who extorted you (not mutually exclusive)			
Narcos		17	25.4
Gangs		12	17.9
Police		54	80.6
Military		14	20.9
Immigration		25	37.3
Other		12	17.9
Country extorted (not mutually exclusive)			
Mexico		52	77.6
Guatemala		45	67.2
Honduras		12	17.9
El Salvador		0	0.0
Colombia		15	22.4
Panama		6	9.0
Nicaragua		14	20.9
Costa Rica		3	4.5
Venezuela		4	6.0
Other		3	4.5
Place of extortion			
Extorted in home country		11	53.7
Extorted during migration (outside home country)		36	29.9
Extorted in both home and outside country		20	16.4
Total number of types extorters	2	1.1	

*65 of the 67 people who endorsed being extorted provided an amount, the average is based on those who provided an amount of extortion paid

Table 3 presents mental health characteristics of those who were extorted versus those who were not. Those who were extorted (n = 67) compared to those who were not (n = 18), had significantly higher PCL-5 scores (mean 26.9 vs 16.9; *p* = 0.02). We also found that those who experienced extortion were significantly more likely to rate their health as excellent, very good, or good (92.3% vs. 66.7%, *p* = 0.005). There were not significant differences by number of ACEs, depression or anxiety symptom severity. (see Table 3). It should be noted that the skewness and kurtosis, as tests of normality, were conducted. The skewness showed acceptable ranges well within the standard -1 to 1, with values ranging from 0.151 to to 0.469. With respect to kurtosis, two variables (GAD7 and the PCL5) fell just out of the -1 to 1 range, with values of -1.344 and -1.123, respectively. The remaining variables ranged from -0.689 to -0.997.

**Table 3:** Mental health characteristics

		Full sample (n = 85)		Not extorted (n = 18)		Extorted (n = 67)		Sig.
		n/μ	%/SD	n/μ	%/SD	n/μ	%/SD	
*Mental Health*								
ACEs score		2.4	1.9	2.2	1.9	2.4	1.8	0.660^a^
GAD7 total score		10.3	7.1	8.9	6.8	10.7	7.2	0.307^b^
PHQ9 total score		10.7	8.0	9.9	8.9	11.0	7.8	0.548^b^
PCL5 total score		25.4	19.3	16.9	20.1	26.9	18.6	**0.016**^b^
Self rated overall health (excellent/very good/good)		72.0	84.7	12	66.7	60	92.3	**0.005**^c^
Self rated QOL (excellent/very good/good)		44	51.8	13	72.2	31	47.7	0.065^c^
Self rated Mental Health (excellent/very good/good)		59	69.4	11	61.1	48	73.8	0.292^c^

Bold values are significance by* p* < 0.05

a = t-test, b = Mann Whitney U, c = Chi-Square

## Discussion

We demonstrate that extortion experiences are exceedingly common amongst immigrants arriving at the southern US border with more than three quarters of those interviewed endorsing at least one extortion experience. Prior research mentions extortion as one of the multiple traumas suffered by Latinx migrants to the US (Vogt 2013; Lusk et al. 2021), however, ours is the first study to our knowledge to describe prevalence rates, amounts paid, and types of extorters mentioned in the description of extoration experiences among a sample of Latinx migrants presenting at the US southern border. For example, prior research identifies patterns of extortion of migrants by police through qualitative research in Mexico (Vogt 2013), but our study is able to provide prevalence estimates of migrants extorted by police throughout the migration journey. The average amount paid from extortion was $804 US dollars per person (in our sample this amounts, in total, to more than $51,000). These costs are acknowledged by participants to be in addition to the large sums often required for smugglers or coyotes to guide migrants through various points of the journey to the US border (Díaz Ferraro et al. 2020). To our knowledge prior research has not quantified the amount of money extorted per immigrant translated to US dollars. Our study suggests that Latinx immigrants often suffer a heavy financial burden via extortion.

Prior work has supported that extortion of small businesses is widely prevalent in Central America and Mexico; (Wolf 2016) small businesses are commonly extorted by gangs (Oxford Analytica 2016). Extortion’s unrelenting fees and financial uncertainly have impoverished Central Americans and devastated the Micro-, Small, and Medium Enterprises (MSMEs), in which a large majority of the population works. Nearly 80% of MSMEs in El Salvador and 70% of Honduras MSMEs reported paying extortion (a large part of the US $737 million Hondurans paid extortionists in 2022). MSMEs pay weekly or monthly fees, with regular non-negotiable increases on top of 20% to 40% interest rate payments for money borrowed from loan shark mafias to meet gang fees (El Heraldo 2016). Such payments have led to mass bankruptcy, including the closure of 40,000 Honduran MSMEs from 1996 to 2016 (Tiempo 2016). Central Americans victimized by extortive threats relocate an average of five times within their countries (Ungar et al. 2022). Thus, it would be reasonable to predict that extortion in our sample would be more prevalent in our participant’s home countries. Instead, almost half of individuals report being extorted *only* during their migration journey, though almost 20% of our sample reported beign extorted both before *and* during migration. Previous research outlines the “systematic” and “inescapable” violence and exploitation that migrants face in Mexico in recent years. Mass movement of people creates opportunities for criminal organizations to collude with local law enforcement in the use of violence to derive a profit from extortion and other crimes (Vogt 2013).

Many participants shared similar details about their extortion experiences. For example, several participants described travelling through Mexico on commercial buses as ticketed passengers and were stopped by police at various points throughout the routes. Non-Mexican passengers were identified by police (through demands of identification or by their spoken accents), often times made to get off the bus, and only allowed to continue their journey after they paid an extortion fee. Many participants told us that police would often coerce them into paying extortion fees through threats of deportation or of being handed over to criminal organizations.

Previous studies also suggest that not all relationships between migrants and smugglers or “facilitators” are exploitative (Sanchez 2017). We agree with this research and believe that this study differs from this work in a couple important ways. First, in our study, the most common type of extorter were police and immigration officials. While it is plausible that some law enforcement personnel would be involved in smuggling activities, we did not find evidence of this in our study but perhaps future studies may provide more insight into that question. Second, home countries were most commonly endorsed for location of extortion experiences. This finding, along with law enforcement being the most common type of extorter, may suggest that corruption of law enforcement officials may be a contributor to extortion experiences being more common in home countries than along the journey. However, we suggest that further research provide a more in-depth look into the relationship between place of occurrence and type of extorter using a larger sample size.

In Mexico and Central American communities, pervasive extortion fills the daily routine of people with fear, danger, and uncertainty (Magaloni et al. 2011). Children as young as eight are forcibly recruited by the *maras* (the name of the two powerful gangs, MS-13 and Barrio 18, that control much of Central America) to keep track of and inform on other residents’ movements, routines, and business transactions (Ungar et al. 2022). In Guatemala, most extortion is carried out by copycats (Martinez 2018)—unknown persons pretending to be mara members—who call up to 100 cell phones a day with extortive demands and threats against family members (Ungar et al. 2022). Worsening such threats is the collaboration of enforcement officials in extortion and other criminality (Fontes 2016). Up to 40% of extortion rackets are run by the police in Honduras, where the police unit handling maras and extortion was dissolved in 2022 due to its widespread human rights abuses (Ungar et al. 2022). Thus, approaches to mitigating these experiences for migrants likely requires institutional support and enforcement of anti-corruption policies among the ranks of law enforcement.

We identify key risk and protective factors associated with extortion experiences. Being pregnant or traveling with a pregnant partner was associated with lower rates of extortion experiences. While prior literature supports that Latinx pregnant migrants experience various forms of violence (Zaman et al. 2023), our findings do not support higher rates of extortion relative to other migrants. Traveling with children was associated with higher rates of extortion. Although individual accounts of extortion of families have been reported in the media ((Human Rights Watch 2021), with specific examples of how children are leveraged in extortion schemes (NBC News 2021; Axios et al. 2021), we find few studies examining the relationship between pregnancy, traveling with children and extortion in general, let alone from Latin America. One possible explanation for higher rates of extortion among those traveling with children may be that parents may be more likely to pay extorters (thus, may be more likely targets) in order to avoid harm to their children. Finally, we find that those who are extorted rate their health better compared to the comparison group. One limitation here is that one’s impression of their own health may not accurately reflect one’s actual health status. It is possible that in order to initiate such a difficult journey, one may need the confidence of having good health. However, we cannot provide a clear explanation for this finding and suggest that future research use larger samples to study this relationship.

Many extortion experiences occurred in Mexico and Guatemala, where extortion of immigrants is common, as these are funnel-like transit countries on the journey to the US; additionally, law enforcement and criminal actors may assume that migrants are vulnerable targets because they are unlikely to report these crimes given varying documentation statuses (Human Rights Watch 2021; Axios et al. 2021; Amnesty International USA 2014; Correa-Cabrera 2023). Starting one’s journey in South America and traveling longer distances were associated with greater extortion risk. Interestingly, reported extortion of migrants in El Salvador, Costa Rica, Venezuela and Panama were relatively low in our study. However, prior work has documented variability in extortion rates by country, and our findings further support that work (Vázquez del Mercado G. et al., Global Initiative Against transnational organized crime 2021).

Extortion in Latin America creates levels of economic poverty, physical danger, social breakdown, and psychological stress (Ungar et al. 2022; Anzola 2016). We hypothesized that individuals experiencing extortion might have higher rates of trauma related symptoms, depression, anxiety, but not ACEs (which, in our adult sample, predate the migration journey).

## Limitations

A limitation of this initial analysis is our small sample size. Another limitation of this study is that our small sample size is due to the fact that more detailed questions about extortion experiences were added to the survey later. Due to our small sample, we conduct bivariate analysis for this study. However, we are aware that many factors may affect mental and physical health. This study recruited participants from a humanitarian respite center, which can possibly introduce selection bias because the respite center mostly provides services to families (i.e. usually adults travelling with children, although there are a small number of adults traveling alone, both female and male). Although this is not ideal, when doing research with a population of newly arrived Latinx immigrants that is very difficult to reach, we are only able to work with participants who are identifiable and with community partners that provide services to this population. This study is not able to capture the experiences of newly arrived immigrants that go undetected upon entry to the US. Future research on how extortion relates to mental health should consider larger sample sizes and the use of multivariate models. Despite these limitations, we believe this exploratory study has useful first time insights related to extortion experiences of Latin migrants and how they may relate to mental health.

## Conclusions

This study of immigrants at the southern US border supports that most individuals have been extorted for money. We document demographics and other characteristics, extortion details, and associated mental health consequences. Additional study of this important topic is needed to shine a light on these commonly experienced migration dangers and identify country risk factors.

## Data Availability

Due to the sensitive nature of this research, we are not able to make data publicly available. However, if there are specific data questions regarding this research, please reach out to the corresponding author Dr. Laura Vargas at laura.x.vargas@cuanschutz.edu.
